# Do Cognitive Behavioural Therapy Interventions Lead to Schema Change in People With Psychosis? A Systematic Review and Meta‐Analysis

**DOI:** 10.1002/cpp.70049

**Published:** 2025-03-03

**Authors:** Nadia Akers, Katherine Berry, Christopher D. J. Taylor

**Affiliations:** ^1^ Division of Psychology and Mental Health, School of Health Sciences The University of Manchester Manchester UK; ^2^ Greater Manchester Mental Health NHS Foundation Trust Manchester UK; ^3^ School of Psychology, Faculty of Science The University of Sheffield Sheffield UK; ^4^ Pennine Care NHS Foundation Trust Ashton‐under‐Lyne UK

**Keywords:** CBT, cognitive behaviour therapy, psychosis, schemas

## Abstract

**Background:**

Negative schemas have been highlighted as important factors in the development and maintenance of psychosis. However, evidence for schema therapy in people with experiences of psychosis and for schema‐specific interventions is lacking for these disabling core beliefs. Cognitive behavioural therapy (CBT) interventions remain a first‐line recommended psychological treatment for psychosis, alongside psychotropic medication. The current review aimed for the first time to investigate if CBT interventions led to schema change in this population.

**Method:**

A systematic search of five databases (PsycINFO, MEDLINE, Embase, CINAHL and Web of Science) identified 19 eligible studies, of which 10 were eligible for inclusion in the meta‐analyses.

**Results:**

A narrative synthesis highlighted the variety in CBT intervention length and focus. A small proportion of studies highlighted schema theory within their therapy rationale and within their subsequent CBT intervention. Meta‐analytic findings demonstrated that participants receiving a CBT intervention experienced a significant reduction in their negative‐self schemas at the end of therapy, compared with control participants.

**Conclusion:**

The findings provide evidence that CBT for psychosis can reduce negative schemas in people with psychosis. The review also offers a rationale for considering schema more explicitly within CBT for psychosis intervention studies and clinical practice.

**Summary**

- There are limited CBT intervention studies for psychosis that include a measure of schema.
- CBT significantly reduces negative schemas in people with psychosis compared to controls.
- Negative schemas may play a crucial role in the development and experience of psychosis.
- Future CBT intervention research would benefit from inclusion of schema measures.

## 1 Introduction

Schemas can be defined as core beliefs about the self, others and the world, which shape an individuals' prediction and interpretation of their environment and guide their responses. Schematic beliefs are proposed to develop as a result of early childhood experiences (Young, Klosko, and Weishaar 2003). Adverse childhood experiences such as trauma, neglect and other factors such as parenting styles are likely to lead to the development of negative or maladaptive schemas. Psychological models of psychosis indicate that negative schematic beliefs play a key role in the development and maintenance of symptoms (Garety et al. 2001). Research also suggests that people with experiences of psychosis are likely to have high levels of negative schemas about themselves and other people (Fowler et al. 2006). It has been suggested that maladaptive negative schemas develop as a result of childhood trauma and stress, which may lead to paranoid interpretations of ordinary experiences (Garety and Freeman 2013). In a review of psychological mediators of psychosis, maladaptive cognitive factors including schemas were also highlighted as mediating factors between childhood adversity and experiences of psychosis in adulthood (Williams et al. 2018). Negative schemas have also been seen to be linked to distress, social functioning and specific symptoms of psychosis, in particular positive symptoms such as paranoia (Sundag et al. 2016; Taylor and Harper 2017).

Although maladaptive schemas about the self and others are developed through negative life experiences, people may also develop alternative, positive schemas through positive life experiences and the strength of schemas can change or shift through therapy (Beck 1979; Dozois and Beck 2023; Taylor, Bee, and Haddock 2017; Young, Klosko, and Weishaar 2003). A growing body of literature also suggests that self and other beliefs may mediate the relationship between attachment style and symptoms of psychosis in adulthood, such as paranoia (Partridge, Maguire, and Newman‐Taylor 2022; Sood, Carnelley, and Newman‐Taylor 2022). Consequently, researchers have highlighted the importance of placing a focus within therapy on lessening the strength of negative schemas and strengthening more positive beliefs about the self, world and others for people with psychosis (Bortolon et al. 2013; Sundag et al. 2016; Taylor and Harper 2017). This may be more challenging in people with predominantly negative life experiences who struggle to access positive schema; therefore, more adaptive schemas must first be developed through therapy (Dozois and Beck 2023). Schema therapy has been designed to directly target negative schemas developed in childhood and to link them to difficulties within the present, to reduce symptoms and distress across a range of diagnoses (Young, Klosko, and Weishaar 2006). Schema therapy uses a schema mode model, using techniques to reduce the strength of negative schemas, such as imagery rescripting, chair work and emotion‐focused techniques, as well as identifying alternative adaptive schemas (Young, Klosko, and Weishaar 2006). Reviews suggest that schema therapy is effective at reducing negative schema and symptoms across mental health diagnoses including depression, anxiety and personality disorders (Bakos, Gallo, and Wainer 2015; Hawke and Provencher 2011; Körük and Özabacı 2018; Taylor, Bee, and Haddock 2017). Despite evidence for the role of maladaptive schemas in psychosis, there is little research investigating schema therapy in this population group.

Cognitive behavioural therapy (CBT) interventions are recommended by UK and international guidance as a preventative therapy for people at risk of developing psychosis and as a first‐line approach for treating first episode and longstanding psychosis alongside antipsychotic medication (Addington et al. 2017; Early Psychosis Guidelines Writing Group 2010; Keepers et al. 2020; National Institute for Health and Care Excellence 2014). In support of this, systematic reviews have demonstrated evidence for symptom change in psychosis following a course of CBT (Bighelli et al. 2018; Burns, Erickson, and Brenner 2014; Lutgens, Gariepy, and Malla 2017; Sitko et al. 2020; Wood et al. 2020). Additionally, CBT appears to improve social and occupational functioning and reduce relapse in people with psychosis as well as reduce transition to psychosis in at‐risk individuals (Bighelli et al. 2021; Frawley et al. 2023; Stafford et al. 2013; Zheng et al. 2022).

Research has suggested that change in maladaptive schemas precedes symptom reduction following CBT for people with OCD and PTSD (Bourdon et al. 2019; Wilhelm et al. 2015). However, it is unclear whether CBT interventions lead to schema change in people with psychosis, despite evidence for the relationship between negative schemas and psychosis (Fowler et al. 2006). Increasing understanding of the processes by which CBT interventions lead to symptom change in psychosis may lead to further development and refinement of interventions. Consequently, in line with theoretical evidence, it would be beneficial to understand whether CBT interventions exert their influence on symptoms through change in schemas. However, there are currently no reviews considering schema change in CBT interventions for psychosis. Evidence is needed to understand whether CBT and third‐wave interventions, offered by mental health services, lead to schema change in people with psychosis. The aim of this review was to consider whether CBT interventions are seen to lead to schema change in people with psychosis.

## 2 Method

### 2.1 Protocol and Registration

The protocol was pre‐registered on PROSPERO CRD42024393409, available from https://www.crd.york.ac.uk/prospero/display_record.php?ID=CRD42024393409

### 2.2 Eligibility Criteria

Study inclusion criteria for the review were as follows: (1) participants included individuals with psychosis, schizophrenia, schizoaffective disorder, delusional disorder, affective psychosis or that were identified as being at high risk for psychosis, self‐reported or diagnosed using ICD or DSM criteria (American Psychiatric Association 2013). The study could include clinical samples recruited via both inpatient (i.e., hospital) and community (e.g., community adult mental health) settings; (2) involved delivering a course of CBT‐focused intervention, including third wave interventions such as compassion‐focused therapy (CFT), acceptance and commitment therapy (ACT) or metacognitive therapy (MT); (3) a randomised controlled trial (RCT), noncontrolled trial, within subject intervention or case series; (4) included a validated quantitative measure of schema change such as the Brief Core Schema Scales (BCSS; Fowler et al. 2006) or the Young Schema Questionnaire (YSQ; Young 1994); and (5) English language text due to language constraints of the research team. Additional inclusion criteria that needed to be met for studies to be included in the meta‐analysis were as follows: (1) RCT design and (2) means and standard deviations were reported of the included schema measure posttherapy in the intervention and control group.

Study exclusion criteria were as follows: (1) participants included with other mental health diagnoses, who were not reported to have psychosis; (2) a systematic review, qualitative study, cross‐sectional study or case study; (3) a measure that aligned more closely to conditional beliefs, such as the Dysfunctional Attitudes Scale (DAS; Weissman and Beck 1978); and (4) Non‐English language full text.

### 2.3 Search Strategy and Data Extraction

Databases searched for this review included PsycINFO, MEDLINE, Embase, CINAHL and Web of Science. These databases were selected as their topic coverage was considered most relevant for the current review. The search strategy was informed by previous systematic reviews in psychosis and CBT to identify relevant search terms (Hazell et al. 2016; Humphrey et al. 2021; Taylor, Bee, and Haddock 2017). Test searches were conducted to ensure the search was gathering all relevant studies without over inclusion of irrelevant ones, leading to further tailoring of the search terms. Limiters were set to include papers published in the English language only. MeSH terms and keyword searches were also conducted for each database where possible. A full list of MeSH terms and keywords can be found in the Supporting Information to this review. The search method is described in Table 1.

**TABLE 1:** Search terms.

String (combined with AND)	Search terms
1	Psychosis or psychoses or schizo* or psychotic or hallucin* or paranoi* or voice hear* or severe mental or serious mental or psychiatric* or unusual belief* or thought disorder*
2	Schema* or belief* or attitude* or BCSS or YSQ
3	Cognitive behavio* or cognitive therapy or behavioural therapy or behaviour therapy or CBT or acceptance and commitment therapy or ACT or compassion focused therapy or CFT or third wave therap* or metacognitive
4	Trial or feasibility or RCT or randomised controlled* or randomized controlled*

The initial screening stage involved a title and abstract search conducted by the primary reviewer on all papers generated. A secondary reviewer conducted a title and abstract search on 10% of papers. Discrepancies were recorded and discussed until an agreement was reached. The reliability of initial screening was assessed through Cohen's kappa. In subsequent screening stages, the primary reviewer screened the full text of eligible papers, which were then discussed and agreed with the research team. Reference and citation lists were also checked for additional papers by the primary reviewer. In instances where multiple references reported the same trial or intervention, the original trial paper was included, and additional papers were excluded to avoid duplication of participant data. Exceptions to this included instances in which schema measure scores were not reported in the original trial paper. In these cases, the paper reporting details of schema measure outcomes was included instead.

A standardised form was used to extract data from the included studies. The extracted information comprised key information about the study, such as study design, therapy type and schema measure.

### 2.4 Quality Assessment

To assess the quality of included studies, an adapted version of the Effective Public Health Practice Project (EPHPP; Supporting Information) Quality Assessment Tool for Quantitative Studies was used (Thomas et al. 2004). As the focus of the current review was on schema change, Section E (Data Collection Methods) of the EPHPP tool was used on the measure(s) of schema only. The original version of the tool allowed non‐RCT studies to be rated as ‘not applicable’ for Sections C (Confounders) and D (Blinding). However, in the current review, studies without a control or comparison group were automatically given a ‘weak’ rating for Sections C and D to acknowledge this limitation in study design. Each component of the EPHPP was given a rating of ‘strong’, ‘moderate’ or ‘weak’, and the study was rated overall. The EPHPP has previously been adapted in other systematic reviews focusing on specific outcomes (Degnan et al. 2018; Humphrey et al. 2021). The quality assessment was conducted by the primary reviewer for all included studies and an independent secondary reviewer rated 50%. Discrepancies were discussed until an agreement was reached.

### 2.5 Data Analysis and Synthesis

Comprehensive meta‐analysis (CMA) Software Version 3 was used for statistical analyses of between‐group effects (Borenstein 2022). Studies employing a between‐subjects' design, for example, RCT studies, were eligible for inclusion within the meta‐analysis. Sample size, postintervention results and follow‐up results (if applicable) for each treatment group were extracted and inputted into CMA for the meta‐analysis. Hedges' *g* effect sizes were calculated in CMA using the standard computational approaches for postintervention or follow‐up means, standard deviations and sample sizes for each study (Borenstein et al. 2009). The last time‐point was used for each study with follow‐up data. Because of anticipated methodological and clinical heterogeneity between included studies, random effects models were conducted as they help to account for studies with considerable heterogeneity and result in more conservative estimates for the meta‐analysis (Field and Gillett 2010). Separate analyses were conducted for schema measure subscales. Cohen's criteria were used for interpretation of the summary effect sizes: 0.2 = small effect, 0.5 = medium effect, 0.8 = large effect (Cohen 1988). For all meta‐analyses conducted, heterogeneity statistics, Cohen's *Q* test and *I* ^2^ statistic, were conducted to consider any statistical inconsistencies in effect sizes, on the basis that *I* ^2^ = 25% = low, 50% = moderate, 75% = high heterogeneity (Higgins and Thompson 2002). Furthermore, Egger's test for funnel plot asymmetry was used to assess publication bias in addition to visual inspection of the funnel plots (Egger et al. 1997) (see Supporting Information). Finally, the one study removed analytic approach was used in CMA to identify whether any of the included studies had a substantial impact on the overall results of the meta‐analysis.

A narrative synthesis was conducted on all studies to describe, compare and contrast intervention characteristics and consideration of schema outcomes. Cochrane guidance provided a framework for the narrative synthesis (Ryan 2013).

## 3 Results

### 3.1 Study Selection

**FIGURE 1:**
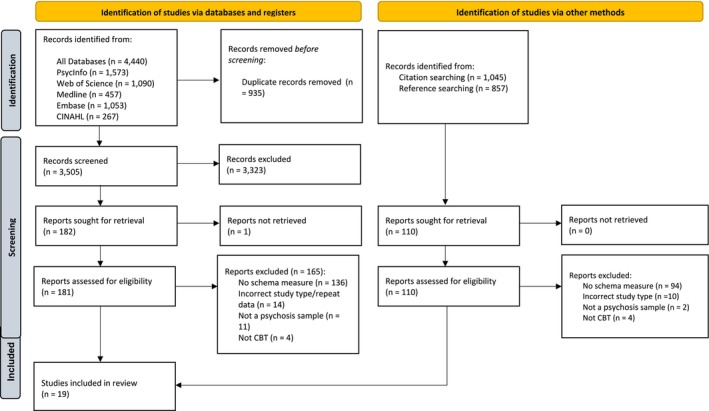
PRISMA 2020 flow diagram (Page et al. 2021).

The database search resulted in a total of 4440 papers. Following duplicate removal, 3505 titles remained to be screened. There was moderate agreement between primary and secondary reviewers at initial screening (*k* = 0.77, *p* < 0.001). A consensus was later achieved following discussion, and 167 papers were ultimately identified as eligible for full‐text screening. Of these, 16 papers met the inclusion criteria. The most common reason for exclusion was that the study did not include a measure of schema outcome (*n* = 136). Three additional papers were identified from reference and citation list searches, resulting in a final selection of 19 papers that met the criteria for inclusion in the review. The full search process is outlined in the PRISMA 2020 diagram in Figure 1 (Page et al. 2021).

### 3.2 Study Characteristics

Key characteristics of the 19 included studies are summarised in Table 2. Of the included studies, 11 used an RCT design. Of the remaining eight studies, five used a within‐subject pretest and posttest design and three used a multiple baseline experimental case series design. These are described as ‘cohort’ studies for the remainder of the review. One study, Hodgekins and Fowler (2010), was a mediation analysis of the results from the ISREP MRC Trial (Fowler et al. 2009). In this instance, the secondary paper from the trial was included as schema outcomes were not reported in the original trial paper.

**TABLE 2:** Summary of study characteristics.

Author and year	Country	Design	Intervention (*n*)	Control (*n*)	Method of delivery	Sessions (*n*)	Therapists	Schema measure	Follow‐up period (n months)
Addington et al. 2023	Canada & USA	Single‐blind RCT	Cognitive behavioural social skills training (70)	Supportive contact (82)	Group	18	Master/doctoral level therapists and cofacilitators	BCSS	12 months posttherapy
Airey, Berry, and Taylor 2023	UK	Multiple baseline experimental case series	Attachment‐focused CBT‐informed intervention; iMAPs (12)	None	Individual	6	Not reported	BCSS	0
Cairns, Kelly, and Taylor 2023	UK	Multiple baseline experimental case series	Attachment‐focused CBT‐informed intervention; iMAPs (5)	None	Individual	6	Trainee clinical psychologist	BCSS	0
Chung et al. 2013	South Korea	Pretest and posttest	CBT (24)	None	Group	12	Principal investigator, trained in CBT for psychosis, with two assistant therapists (clinical psychologist and senior psychiatric resident)	BCSS	End of therapy only
Forkert et al. 2022	UK	Pretest and posttest	Compassionate imagery intervention (12)	None	Individual	4	Trainee clinical psychologist	BCSS	1 month posttherapy
Freeman et al. 2014	UK	Randomised controlled evaluation	Brief CBT (15)	Standard care (15)	Individual	6	Clinical psychologists	BCSS	1 month posttherapy
Freeman et al. 2016	UK	Pretest and posttest	CBT (12)	None	Individual	9–24	Clinical psychologists	BCSS	0
Freeman et al. 2021	UK	Single‐blind RCT	CBT for psychosis; The Feeling Safe Programme (64)	Befriending (66)	Individual	Approx. 20	Clinical psychologists	BCSS	6 months posttherapy
Garety et al. 2008	UK	Multicentre RCT	CBT (133)	Treatment as usual or a family intervention (168)	Individual	M = 14.4	Clinical psychologists	BCSS	12 months posttherapy
Garety et al. 2021	UK	Single‐blind RCT	CBT for psychosis (181)	TAU (180)	Individual	8	Doctoral Level Psychologists	BCSS	3 months posttherapy
Hagen, Nordahl, and Grawe 2005	Norway	Pretest and posttest	CBT (19)	None	Group	16	Psychologists	YSQ‐SF	4 months posttherapy
Hayward et al. 2021	UK	3‐arm parallel group feasibility RCT	Brief guided self‐help CBT; GiVE Intervention + TAU (26)	Supportive counselling + TAU or TAU alone (27)	Individual	8	Assistant Psychologists	BCSS	3 months posttherapy
Hazell et al. 2018	UK	Single‐blind RCT	Guided self‐help CBT; GiVE Intervention (14)	TAU (14)	Individual	8	Clinical psychologists	BCSS	0
Hodgekins and Fowler (2010)	UK	Single‐blind RCT	Social recovery–focused CBT (35)	TAU (42)	Individual	M = 12	CBT therapists or case managers, under supervision of CBT therapists	BCSS	0
Mehl et al. 2021	Germany	Single‐blind RCT	Emotion‐focused CBT (35)	TAU + wait list (29)	Individual	20	Clinical psychologists	BCSS	12 months posttherapy
Pot‐Kolder et al. 2018	Netherlands	Single‐blind RCT	Virtual reality–based CBT (58)	TAU (58)	Individual	16	Psychologists	BCSS	3 months posttherapy
Randal et al. 2016	UK	Pretest and posttest	Mindfulness‐based CBT (21)	None	Group	8	CBT therapist and trained mindfulness practitioner	BCSS	0
Taylor et al. 2020	UK	Multiple baseline experimental case series	CBT‐informed intervention; iMAPS (5)	None	Individual	6	Clinical psychologist	BCSS YSQ–SF SMI	0
Waite et al. 2023	UK	Single‐blind RCT	CBT‐informed SleepWell intervention (21)	TAU (19)	Individual	8	Clinical psychologists	BCSS	6 months posttherapy

Abbreviations: BCSS: Brief Core Schema Scales (Fowler et al. 2006); CBT: cognitive behaviour therapy; GiVE: Guided self‐help cognitive behaviour Intervention for VoicEs; iMAPS: IMAgery focused therapy for persecutory delusions in Psychosis; RCT: randomised controlled trial; SMI: Schema Mode Inventory (Lobbestael et al. 2010); TAU: treatment as usual; YSQ‐SF (Young 1998): Young Schema Questionnaire–Short Form.

Total study sample sizes ranged from 5 to 362. Of the studies reporting between‐group differences, the CBT intervention group sample sizes ranged from 14 to 181 at baseline. Participant diagnoses across studies included schizophrenia, schizoaffective disorder, schizophreniform disorder, non‐affective psychosis, affective psychosis, psychotic disorder, psychotic disorder not otherwise specified, delusional disorder and ‘schizophrenia spectrum disorders’. Two studies included a sample of individuals who were considered ‘at risk for psychosis’, based on the Structured Interview for Psychosis‐risk Syndromes (McGlashan, Walsh, and Woods 2010) and the Structured Clinical Interview for DSM‐5 (First 2014) or the Comprehensive Assessment of At‐Risk Mental States (CAARMS; Yung et al. 2005). Another study included participants who were hearing voices based on the Hamilton Program for Schizophrenic Voices Questionnaire (Van Lieshout and Goldberg 2007). One study included participants meeting the criteria for an early intervention in psychosis service in the United Kingdom. A single study included participants with comorbid depression (Hagen, Nordahl, and Grawe 2005).

Four studies used a group format for the CBT intervention, ranging from 8 to 12 weeks (Addington et al. 2023; Chung et al. 2013; Hagen, Nordahl, and Grawe 2005; Randal et al. 2016). An individual approach was adopted by the remaining 15 studies, varying from four to 20 sessions. Two studies incorporated guided self‐help in the form of additional self‐help workbooks to be completed during the therapy (Hayward et al. 2021; Hazell et al. 2018). Three studies drew on technology to support their CBT intervention, including a digitally supported CBT intervention that combined individual sessions with a digital manual (Garety et al. 2021), a virtual reality–based intervention comprising exposure to virtual social environments (Pot‐Kolder et al. 2018) and an intervention delivered via telehealth (Cairns, Kelly, and Taylor 2023). Five included studies provided an adapted CBT intervention. The most significant adaptation involved the merging of social skills training (SST) with CBT (Addington et al. 2023). SST is an evidence‐based treatment in its own right and has previously been combined with CBT to offer cognitive‐behavioural social skills training (CBSST) for people with schizophrenia (Dixon et al. 2010; Granholm, McQuaid, and Holden 2016). Other included studies adapted their CBT interventions to incorporate additional elements based on psychological theory relating to psychosis (Freeman et al. 2021; Garety et al. 2008; Hodgekins and Fowler 2010; Mehl et al. 2021; Waite et al. 2023). These additions included a focus on emotion regulation, social recovery, self‐beliefs, sleep dysfunction, worry and safety, and one study targeted key aspects of relapse. Others used imagery, mindfulness, compassion and attachment‐focused approaches (Airey, Berry, and Taylor 2023; Cairns, Kelly, and Taylor 2023; Forkert et al. 2022; Randal et al. 2016; Taylor et al. 2020).

All but one study used the BCSS to measure schema outcome (Fowler et al. 2006). Other measures included the Young Schema Questionnaire–Short Form (YSQ‐SF; Young 1998) and the Schema Mode Inventory (SMI; Lobbestael et al. 2010). Twelve studies followed participants up after the end of therapy, ranging from 1 to 12 months posttherapy. Three of the included RCT studies provided a rationale for the therapy involving the need to target negative beliefs or schemas in therapy due to evidence of a putative causal mechanism for experiences of psychosis, in particular paranoia or persecutory delusions (Freeman et al. 2021; Freeman et al. 2014; Mehl et al. 2021). A fourth included RCT study highlighted the importance of increasing positive self‐concept (Hodgekins and Fowler 2010). However, only one of these four studies included schema as a primary outcome measure (Freeman et al. 2014). Out of the 11 included RCTs, six included schemas as a secondary outcome measure (Addington et al. 2023; Garety et al. 2021; Hayward et al. 2021; Hodgekins and Fowler 2010; Pot‐Kolder et al. 2018; Waite et al. 2023). Others included schemas as a measure of mediation (Freeman et al. 2021; Mehl et al. 2021), a proposed mechanism of action (Hazell et al. 2018) or a measure of therapy process (Garety et al. 2008). One RCT study did not report on data for schema outcomes and did not provide these when contacted and therefore could not be included in the meta‐analysis (Garety et al. 2008). However, Garety et al. (2008) did not report any significant changes in the predicted direction of schema as a treatment mediator. Ten studies were therefore included in the meta‐analyses. Schema measures were not included as a primary outcome within any of the cohort studies; however, this reflected the fact that most were feasibility and acceptability studies that did not specify a primary outcome.

### 3.3 Quality Appraisal

The overall quality of the 19 studies included in the review was weak. However, this picture was skewed by the eight cohort studies, which were each rated as weak for confounders and blinding because of limitations in their study design. The cohort studies were not eligible for inclusion in the meta‐analyses. In considering only the RCT studies, the overall quality of studies was moderate, with three receiving a strong rating overall. The RCT studies were rated strong in design, and all but one of the RCT studies were evaluated as strong for confounders as stratification of relevant confounders was applied in the design or included as a covariate during analysis. However, only three studies were rated as strong for blinding, reporting that as well as the outcome assessors' being blind to participants' allocation status, participants were not aware of the research question (Addington et al. 2023; Freeman et al. 2021; Freeman et al. 2014). All included studies were rated as strong for their schema data collection measure as all studies used the BCSS, YSQ‐SF and/or SMI, which have all previously been described as having good construct validity and internal consistency (Fowler et al. 2006; Lobbestael et al. 2010; Young 1998). Most studies reported withdrawals and dropouts in terms or numbers and reasons per group and had a follow‐up rate of 80% or higher. There was excellent agreement between reviewers (*k* = 0.80, *p* < 0.001). The results of the quality appraisal are reported in Table 3.

**TABLE 3:** Quality appraisal of included studies.

Author	Selection bias	Design	Confounders	Blinding	Data collection—Schema	Withdrawals and dropouts	Global rating
Addington et al. 2023	Weak	Strong	Strong	Strong	Strong	Strong	Moderate
Airey, Berry, and Taylor 2023	Moderate	Moderate	Weak	Weak	Strong	Moderate	Weak
Cairns, Kelly, and Taylor 2023	Weak	Moderate	Weak	Weak	Strong	Strong	Weak
Chung et al. 2013	Weak	Moderate	Weak	Weak	Strong	Strong	Weak
Forkert et al. 2022	Moderate	Moderate	Weak	Weak	Strong	Strong	Weak
Freeman et al. 2014	Moderate	Strong	Strong	Strong	Strong	Strong	Strong
Freeman et al. 2016	Moderate	Moderate	Weak	Weak	Strong	Strong	Weak
Freeman et al. 2021	Moderate	Strong	Strong	Strong	Strong	Weak	Moderate
Garety et al. 2008	Weak	Strong	Strong	Moderate	Strong	Weak	Weak
Garety et al. 2021	Moderate	Strong	Strong	Moderate	Strong	Weak	Moderate
Hagen, Nordahl, and Grawe 2005	Moderate	Moderate	Weak	Weak	Strong	Weak	Weak
Hayward et al. 2021	Moderate	Strong	Strong	Moderate	Strong	Weak	Moderate
Hazell et al. 2018	Moderate	Strong	Weak	Moderate	Strong	Strong	Moderate
Hodgekins and Fowler 2010	Weak	Strong	Strong	Moderate	Strong	Strong	Moderate
Mehl et al. 2021	Moderate	Strong	Strong	Moderate	Strong	Moderate	Strong
Pot‐Kolder et al. 2018	Weak	Strong	Strong	Moderate	Strong	Moderate	Moderate
Randal et al. 2016	Moderate	Moderate	Weak	Weak	Strong	Moderate	Weak
Taylor et al. 2020	Moderate	Moderate	Weak	Weak	Strong	Strong	Weak
Waite et al. 2023	Moderate	Strong	Strong	Moderate	Strong	Strong	Strong

### 3.4 Meta‐Analysis

#### 3.4.1 End of Therapy

**FIGURE 2:**
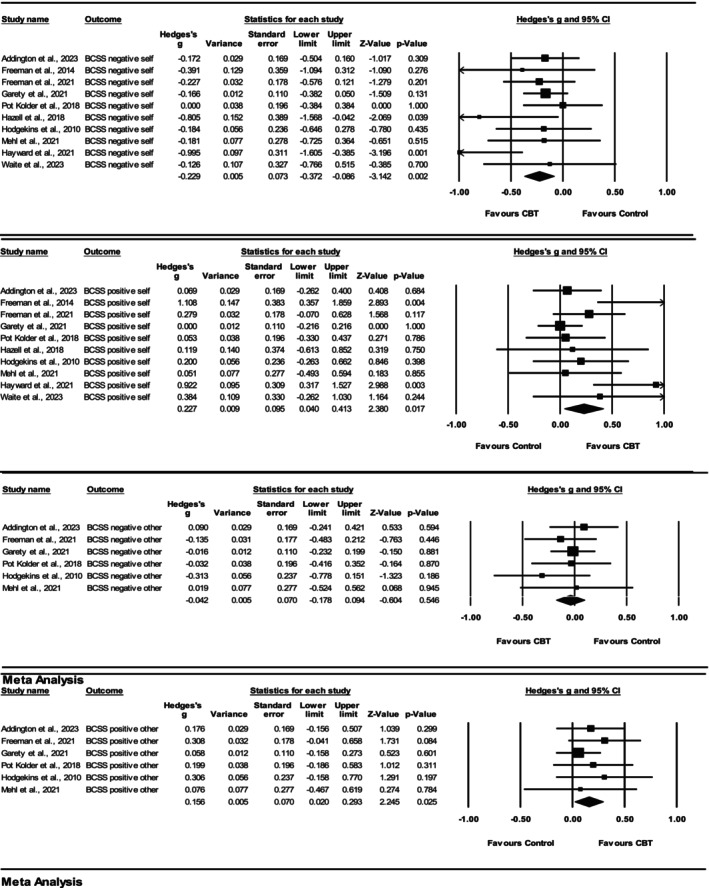
Forest plots demonstrating change in BCSS outcomes for CBT versus control groups at the end of therapy.

Postintervention group summary effects for each study included in the meta‐analysis are displayed in Figure 2, per BCSS subscale (negative‐self, positive‐self, negative‐other and positive‐other).

#### 3.4.2 BCSS Negative‐Self

Ten studies met the criteria for the meta‐analysis of the BCSS negative‐self subscale at the end of therapy, and a significant summary effect was observed (Hedges' *g* = −0.23 [95% CI −0.40 to −0.09, *p* = 0.00]). The meta‐analytic results indicated that, on average, negative‐self schemas reduced significantly more for participants who were delivered CBT compared with controls at the end of therapy. The heterogeneity analysis indicated low levels of statistical heterogeneity: *Q* (9) = 10.39, *p* = 0.32, *I* ^2^ = 13.41% (Higgins and Thompson 2002). The funnel plot indicated possible asymmetry and Egger's test for a regression intercept resulted in a one‐tailed *p*‐value of 0.05. This indicates that the BCSS negative‐self subscale results may have been influenced by publication bias. The one study removed analyses conducted in CMA software did not highlight any single study to be exerting excessive influence or alter the significance of the estimated summary effect.

#### 3.4.3 BCSS Positive‐Self

Ten studies met the criteria for the meta‐analysis of the BCSS positive‐self subscale at the end of therapy, and a significant summary effect was observed (Hedges' *g* = 0.23 [95% CI 0.04 to 0.41, *p* = 0.02]). The meta‐analytic results indicated that, on average, positive‐self schemas increased significantly more for participants who were delivered CBT compared with controls at the end of therapy. The heterogeneity analysis indicated moderate levels of statistical heterogeneity; *Q* (9) = 16.04, *p* = 0.07, *I* ^2^ = 43.88%. The funnel plot indicated possible asymmetry, and Egger's test for a regression intercept provided a *p*‐value of 0.01, suggestive of possible publication bias. One study removed analysis indicated that removing Hayward et al. (2021) would have resulted in a reduced summary effect size of 0.15 and a non‐significant result (*p* = 0.06).

#### 3.4.4 BCSS Negative‐Other

Six studies met the criteria for the meta‐analysis of the BCSS negative‐other subscale at the end of therapy, and a non‐significant summary effect was observed (Hedges' *g* = −0.04 [95% CI −0.18 to 0.09, *p* = 0.55]). Therefore, the meta‐analytic results indicated that, on average, negative‐other schemas did not reduce significantly more for participants who were delivered CBT compared with controls at the end of therapy. The heterogeneity analysis indicated an *I* ^2^ value of 0% as the *Q* value was less than the degrees of freedom: *Q* (5) = 2.31. This is often seen in meta‐analyses with a small number of studies and therefore limits the interpretability of the *I* ^2^ statistic (von Hippel 2015). The funnel plot did not reveal any significant asymmetry, and Egger's test for a regression intercept resulted in a one‐tailed *p*‐value of 0.25. This indicated that the BCSS negative‐other subscale results were unlikely to be influenced by publication bias. One study removed analyses did not highlight any single study to be exerting excessive influence or alter the significance of the estimated summary effect.

#### 3.4.5 BCSS Positive‐Other

Six studies met the criteria for the meta‐analysis of the BCSS positive‐other subscale at the end of therapy, and a significant summary effect was observed (Hedges' *g* = 0.16 [95% CI −0.02 to 0.29, *p* = 0.03]). Therefore, the meta‐analytic results indicated that, on average, positive‐other schemas increased significantly more for participants who were delivered CBT compared with controls at the end of therapy. The heterogeneity analysis indicated an *I* ^2^ value of 0% as the *Q* value was less than the degrees of freedom: *Q* (5) = 2.08, reducing interpretability. The funnel plot revealed possible asymmetry; however, Egger's test for a regression intercept resulted in a one‐tailed *p*‐value of 0.12. This indicated that the BCSS positive‐other subscale results were unlikely to be influenced by publication bias. One study removed analyses indicated that removing Freeman et al. 2021 would have resulted in a non‐significant summary effect (Hedges' *g* = 0.13 [95% CI −0.02 to 0.28, *p* = 0.09]).

#### 3.4.6 Follow‐Up

**FIGURE 3:**
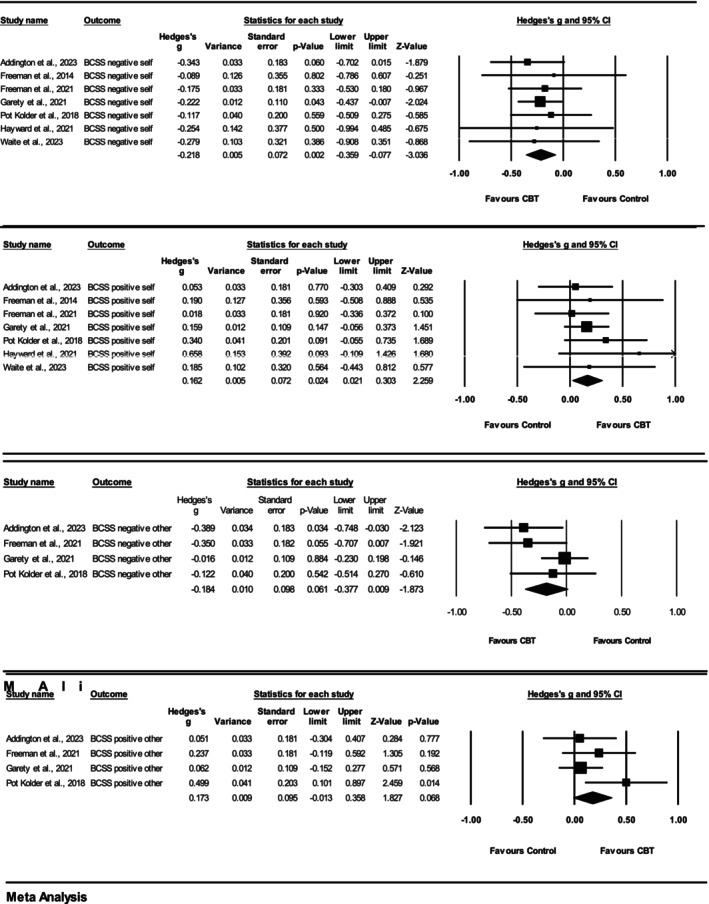
Forest plots demonstrating change in BCSS outcomes for CBT versus control groups at follow‐up.

Follow‐up group summary effects for each study included in the meta‐analysis are displayed in Figure 3, for each BCSS subscale (negative‐self, positive‐self, negative‐other and positive‐other), and summarised below.

#### 3.4.7 BCSS Self Subscales

Seven studies met the criteria for the meta‐analyses of the BCSS negative‐self and positive‐self subscales at follow‐up, and significant summary effects were observed, replicating the end‐of‐therapy findings. The meta‐analytic results therefore indicated that, on average, negative‐self schemas reduced, and positive‐self schemas increased significantly more for participants who were delivered CBT compared with controls at follow‐up. The heterogeneity analyses for both BCSS negative‐self (*Q* (6) = 0.96) and BCSS positive‐self (*Q* (6) = 3.40) indicated *I* ^2^ values of 0% as the *Q* value was less than the degrees of freedom, reducing interpretability. Funnel plots did not reveal any significant asymmetry for either subscale, and Egger's test for a regression intercept resulted in non‐significant one‐tailed *p*‐values: 0.41 and 0.18, respectively. This indicated that the BCSS negative‐ and positive‐self subscale results were unlikely to be influenced by publication bias. Although one study removed analyses did not highlight any single study to be exerting excessive influence or alter the significance of the estimated summary effect of BCSS negative‐self, two studies were highlighted for BCSS positive‐self. Removing Garety et al. (2021) (Hedges' *g* = 0.17 [95% CI −0.02 to 0.35, *p* = 0.08]) and Pot‐Kolder et al. (2018) (Hedges' *g* = 0.14 [95% CI −0.01 to 0.29, *p* = 0.08]) would have resulted in non‐significant summary effects for the BCSS positive‐self at follow up.

#### 3.4.8 BCSS Other Subscales

Four studies met the criteria for the meta‐analyses of BCSS negative‐ and positive‐other subscales at follow‐up. Non‐significant summary effects were observed at follow‐up. This replicated the BCSS negative‐other findings at the end of therapy. The meta‐analytic results, therefore, indicated that, on average, negative‐ and positive‐other schemas did not change significantly more for participants who were delivered CBT compared with controls at follow‐up. The heterogeneity analysis indicated low to moderate levels of statistical heterogeneity: *Q* (3) = 4.42, *p* = 0.22, *I* ^2^ = 32% and *Q* (3) = 4.13, *p* = 0.10, *I* ^2^ = 27%, respectively. Inspection of funnel plots did not reveal any significant asymmetry, and Egger's test for a regression intercept was non‐significant. One study removed analyses revealed that removing Garety et al. (2021) would have resulted in a significant summary effect for both BCSS negative‐other (Hedges' *g* = −0.30 [95% CI −0.51 to 0.08, *p* = 0.01]) and BCSS positive‐other (Hedges' *g* = 0.25 [95% CI 0.00 to 0.50, *p* = 0.05]), at follow‐up.

### 3.5 Narrative Synthesis

Eight included studies used a cohort design and were not eligible for inclusion in the meta‐analysis. Only one cohort study reported a statistically significant change in schemas between pretherapy and posttherapy, finding that negative‐self and positive‐other schemas improved at the end of therapy (Chung et al. 2013). Other studies, however, did not find a significant change in schema pretherapy and posttherapy (Hagen, Nordahl, and Grawe 2005; Randal et al. 2016; Taylor et al. 2020). When statistical significance was not considered, five cohort studies reported improvements in the sample's schema scores at the end of therapy (Airey, Berry, and Taylor 2023; Cairns, Kelly, and Taylor 2023; Forkert et al. 2022; Freeman et al. 2016; Taylor et al. 2020). Of these, two studies commented on the effect sizes of these changes that ranged from small to large (Forkert et al. 2022; Taylor et al. 2020). The results of the cohort studies show a mixed picture of schema change in comparison with the RCT studies described in the meta‐analysis. It may be important to highlight that the included cohort studies had much smaller sample sizes, with the largest sample comprising 24 participants, compared with the largest RCT sample, which consisted of 181 intervention participants. Additionally, the weaker study designs used by the cohort interventions may explain the limited results compared with RCTs, which used a more robust design. The quality assessment highlights this discrepancy in study design as all the cohort studies received a weak rating, whereas the RCTs were moderate overall. Therefore, more confidence may be placed on the results of the meta‐analysis than considering individual cohort studies, most of which focused on the feasibility and acceptability of their intervention rather than stringent design and control.

## 4 Discussion

The aim of the current review was to consider whether CBT‐informed interventions lead to a change in schema outcomes in people with experiences of psychosis. Database searches and screening revealed 19 eligible studies including 11 RCTs and eight cohort intervention studies. Ten studies were identified as eligible for inclusion in the meta‐analysis.

The results from the meta‐analysis of included RCT studies demonstrated that across the 10 included trials, participants who were allocated to a CBT intervention experienced a significant improvement in their negative‐ and positive‐self schemas at the end of therapy, compared with control participants. These improvements were seen to be sustained at follow‐up. Participants who received a CBT intervention were also seen to experience a significant increase in positive‐other schemas at the end of therapy, compared with those who received a control condition. However, this effect on positive‐other schemas was not sustained at follow up, and CBT interventions were not seen to have a significantly different effect on negative‐other schemas at the end of therapy or follow‐up. These results indicate that CBT interventions lead to improvements in both positive‐ and negative‐self schemas in people with experiences of psychosis, which are sustained even after the therapy has ended. Additionally, CBT interventions may lead to improvements in positive‐other schemas; however, the findings indicate a temporary nature to these increases. Consequently, CBT interventions may lead to more consistent change in self‐schemas compared with schemas relating to other people. It could be theorised that participants had fewer positive alternative schemas relating to others, due to repeated, confirmatory negative life events involving other people. Psychosis has been seen to be linked to childhood adversities and traumatic life events with the intention to harm (Moriyama et al. 2018; Varese et al. 2012). Thus, reducing negative‐other schemas through CBT may be more difficult if individuals have experienced repeated harm from others, and a longer intervention may be required to result in schema change. Evidence of schema therapy for personality disorders, for example, suggests greater effectiveness with longer courses of therapy (Jacob and Arntz 2013). In contrast, individuals may have greater access to positive‐self schemas, which makes these more easily accessed when contradictory evidence for negative‐self schemas is presented during therapy, and therefore, change is achieved more quickly with a briefer CBT intervention. Furthermore, negative‐other schemas are suggested to predict persecutory ideation independently, whereas negative‐self schemas are suggested to be mediated by negative affect (Galbraith et al. 2014). Negative‐other beliefs may be more challenging to shift in people who experience paranoia and persecutory delusions. The results from included cohort studies, however, indicated mixed results of CBT interventions on all schema outcomes with only one reporting significant improvement in negative‐self and positive‐other schemas at the end of therapy in their sample (Chung et al. 2013). However, cohort studies had smaller sample sizes and weaker study designs compared with studies included in the meta‐analysis. Hence, results need to be replicated in larger trial designs with a control group and assessors blinded to allocation status. The mixed results could also be explained by the variety of techniques used across the studies, from traditional CBT approaches such as cognitive restructuring to adapted approaches making use of imagery rescripting to target negative schemas associated with negative life events. Overall, the results of this review highlight that, although most CBT interventions do not explicitly focus on changing individuals' negative schemas directly, schemas may be targeted through different uses of language, by considering beliefs about self and others. Through working with negative automatic thoughts, assumptions and maintenance cycles, shifts in core beliefs or schemas may also be a result of cognitive and behavioural change.

The results from the meta‐analysis revealed low to medium levels of statistical heterogeneity, suggesting that effect sizes may have varied to some extent across the included studies. However, for the meta‐analyses of BCSS negative‐self at the end of therapy and the BCSS positive‐other at follow‐up, this variation is likely to have been negligible. The variation in effect sizes that was seen may be accounted for by methodological differences in the included studies. For example, the included studies varied in terms of sample size, intervention and follow‐up length. It is also possible that the statistical heterogeneity could have been impacted by characteristics of the sample that varied between studies, such as diagnosis, chronicity or service setting. The overall quality of studies included within the meta‐analysis was moderate, increasing the confidence that can be placed on the meta‐analytic results.

Seven out of the 19 included studies provided a rationale for therapy that highlighted maladaptive or negative schemas, or self‐concept as key contributors to experiences of psychosis such as persecutory delusions. Nevertheless, despite this therapy rationale, only one study included schema as a primary outcome, although three of the cohort studies did not rank their included measures (Freeman et al. 2014). Schema outcomes were frequently included as a mediator or mechanism of action, suggesting that CBT interventions are intended to target symptoms or other primary outcomes through the reduction of negative schemas. Therefore, it is possible that CBT interventions work to alleviate symptoms of psychosis through the improvement of negative schemas; however, no studies directly tested this. One cohort study did evaluate whether their intervention would result in schema change, with an indirect decrease in persecutory delusions (Taylor et al. 2020). However, as this was a feasibility and acceptability study, with only five participants, this process was not fully tested using any mediation or path analyses.

All but one of the studies included in this review used the BCSS (Fowler et al. 2006). The YSQ‐SF and SMI were also used, less frequently (Lobbestael et al. 2010; Young 1998). Consistency across studies in the use of the BCSS to assess schema was beneficial in aiding comparisons across studies. A strength of the BCSS is that it provides subscale scores of positive‐ and negative‐self and ‐other schemas, meaning it is able to demonstrate whether therapy results in a reduction in negative schemas in addition to an increase in positive schemas. The BCSS also retains simplicity by merging schemas together into positive and negative scores. In contrast, the YSQ‐SF provides a more detailed overview of schematic beliefs, including 90 items, representing 18 early maladaptive schemas. However, because of its comprehensive nature and length, it may be less likely to be selected within trials with a battery of outcomes. The BCSS could therefore be argued to be a less nuanced measure of schema than the YSQ‐SF. During the screening stage of the current review, it became clear that some studies that claimed to measure schema were actually using a measure of conditional beliefs or dysfunctional assumptions, such as the DAS, which identify ‘if … then’ beliefs and are a different but similar concept to schema (Weissman and Beck 1978). Padesky (1994) highlighted a clear distinction between schemas and conditional beliefs, and although the DAS does measure beliefs, these differ from schema in that they are based on conditional criteria rather than fixed core beliefs about the self and others. They are also thought to be evaluated and changed through different therapeutic processes (Padesky 1994). It was important to ensure that the current review did not confuse the two concepts. The difference between schema and conditional beliefs is, consequently, an important distinction that needs to be made clear within future literature.

### 4.1 Limitations

This review has several limitations that need to be considered when interpreting the results. Firstly, limiting studies to those written in the English language meant that possible intervention trials conducted in non‐English speaking countries may have been missed. Initial searches highlighted that there were limited RCTs of CBT for psychosis that collected schema outcome data. Consequently, the incorporation of other study designs was necessary to obtain a clearer picture of the literature to date on this topic. However, the resulting meta‐analyses were conducted on a small number of studies. Because of the variety in the data collected across studies, the meta‐analyses of ‘other‐schema’ and follow‐up data were limited by an even smaller number of studies. Moreover, summary effects of the meta‐analyses of BCSS other schemas at the end of therapy and the BCSS self‐schemas at follow‐up should be interpreted cautiously, due to lack of knowledge about the statistical heterogeneity of included studies.

Some of the included interventions varied considerably in length from each other. For example, the compassionate imagery intervention conducted by Forkert et al. (2022) lasted only four sessions, compared with the longest included studies that lasted for 20 sessions (Freeman et al. 2016; Garety et al. 2008; Mehl et al. 2021). The briefest study included in the meta‐analysis was six sessions over an 8‐week period (Freeman et al. 2014). Follow‐up data included in the meta‐analyses also varied in length as Addington et al. (2023) collected follow‐up data up to 12 months after the end of therapy, whereas Freeman et al. (2014) collected data 1 month after their therapy had ended. This should be taken into consideration in interpreting the results as longer courses of therapy may be required to observe significant schema change. It is important to highlight that in practice, CBT is used to describe a range of interventions delivered by practitioners with variable accreditations and levels of experience. This was also the case within the included studies, with therapist qualifications including clinical psychologists with extensive CBT experience, trainee clinical psychologists, assistant psychologists and CBT therapists. Similarly, included studies tailored their interventions in different ways depending on study goals. This may have contributed to the variability in results, and in practice, it is challenging to isolate key factors within therapy that may be driving the change.

### 4.2 Future Research

To further understand the impact of CBT interventions on schemas in people with experiences of psychosis, further RCTs of CBT interventions, with large psychosis samples measuring schema outcomes, are required. Larger sample sizes would increase the power of studies to be able to detect schema effects. Furthermore, including self and other schema outcomes as measures of the therapy process or mechanism in CBT trials would provide important understanding of how CBT interventions exert their influence on symptoms and functioning. If theoretical models are accurate, then achieving schema change would result in less activation of conditional beliefs and dysfunctional assumptions and therefore fewer symptoms. The quality appraisal indicated that RCTs investigating schemas as a mechanism of change in CBT for psychosis would benefit from controlling confounders, ensuring that participants are blind to the research question, as well as in‐depth reporting of withdrawals and dropouts to increase study quality.

If early maladaptive schemas are suggested to mediate symptoms and functioning in people with psychosis, further research is needed to consider the effect of schema therapy on schema change in psychosis. Evidence is currently limited despite suggestions that schema therapy reduces symptoms in other disorders (Bakos, Gallo, and Wainer 2015; Hawke and Provencher 2011; Taylor, Bee, and Haddock 2017). Without this evidence, there is no rationale for services to change their therapeutic strategies for people with experiences of psychosis. Therefore, feasibility trials of schema therapy for psychosis would be a first step to generating this understanding and may help contribute to service change and implementation of schema therapy.

Future research may also benefit from focusing on the availability of both positive‐ and negative‐self and ‐other schema in people with psychosis. This would contribute to knowledge on processes within the development and maintenance of psychosis, as well as priorities for therapy. Bringing about schema change in people without alternative adaptive schemas may be a greater challenge, and thus, further research into the link between traumatic life events, schemas and schema change in people with psychosis may help to increase understanding of these processes within this population.

### 4.3 Clinical Implications

This review highlights the importance of considering schema as an outcome within CBT for psychosis. This might involve identifying key negative schemas with clients in CBT assessment through clinical interview and/or schema measures and incorporating them within the psychological formulation, ensuring that time is given to identify schemas and core beliefs that may be driving and maintaining difficulties. Given the prevalence of insecure attachments in people with psychosis, previous reviews have also advocated for this focus on self and other beliefs within psychological therapy for psychosis (Partridge, Maguire, and Newman‐Taylor 2022; Sood, Carnelley, and Newman‐Taylor 2022). The results of this review indicate that CBT for psychosis has a more consistent impact on self‐schemas; therefore, the tailoring of therapy to focus on schemas relating to other people, and developing more adaptive other‐schemas, may increase the benefit for individuals with key negative‐other schemas exerting influence within their formulation. This might involve the use of imagery or drawing on schema therapy techniques, such as schema mode dialogue work, which, due to its experiential nature, is effective in activating a client's schemas and can be used to rescript difficult memories (Rafaeli, Bernstein, and Young 2010). Additionally, including schema change outcomes in practice would help to evidence schema change from CBT interventions within services and may help contribute to future service development. High quality and quantity of evidence is needed to result in service change, in addition to research focused on the implementation and integration of new developments within mental health services (Proctor et al. 2009). Training for therapists regarding the importance of considering schemas within therapy may be beneficial for people with psychosis. Further research and evidence in practice is needed to justify funding this.

## 5 Conclusion

The findings from the current review demonstrate evidence for schema change as an outcome from CBT interventions, particularly regarding negative‐ and positive‐self schemas. This supports theories suggesting that negative schematic beliefs play a key role in the development and maintenance of symptoms in people with experience of psychosis (Garety et al. 2001). However, the review findings should be interpreted with caution due to the inclusion of a few high‐quality studies with RCT designs. Further intervention studies are required, with large samples and a control condition, to increase confidence in the findings of this review. Despite this, the results of the current review highlight the importance of further research considering both CBT and schema therapy, in addition to strategies to foster the implementation of a schema focus within services for people with experiences of psychosis.

## Supporting information


**Table S1** MeSH terms and keywords.
**Figure S1** Funnel plots.

## Data Availability

Data sharing not applicable ‐ no new data generated, or the article describes entirely theoretical research.
