# Supplementary material for: Do Cognitive Behavioural Therapy Interventions Lead to Schema Change in People With Psychosis? A Systematic Review and Meta‐Analysis
Source: Clin Psychol Psychother. 2025 Mar 3;32(2):e70049. doi: 10.1002/cpp.70049 (PMC11876722; doi:10.1002/cpp.70049)
Supplement: Supplementary file 1 — Table S1 MeSH terms and keywords. Figure S1 Funnel plots. [file CPP-32-e70049-s001.docx]

# **Supplementary Material**

### Supplementary Table 1: MeSH Terms and Keywords

| Database | Subject Heading or MeSH Term | | | |
| --- | --- | --- | --- | --- |
|  | String 1 | String 2 | String 3 | String 4 |
| PsycInfo | Affective Psychosis  Paranoid Psychosis  Psychosis  Schizophrenia  Brief Psychotic Disorder  Visual Hallucinations  Auditory Hallucinations  Hallucinations  Paranoia  Schizoaffective Disorder  Mental Disorders  Serious Mental Illness  Chronic Mental Illness  Psychiatric Patients  Delusions  Thought Disorders | Schema  Adult Attitudes  Attitudes  Attitude Change  Attitude Measures | Cognitive Behavior Therapy  Cognitive Therapy  Behavior Therapy  Acceptance and Commitment Therapy | Clinical Trials  Treatment Effectiveness Evaluation |
| MEDLINE | Psychotic Disorders  Affective Disorders  Psychotic  Hallucinations  Delusions  Schizophrenia  Paranoid Disorders  Schizophrenia  Mental Disorders | Attitude | Cognitive Behavioral Therapy  Behaviour Therapy  Acceptance and Commitment Therapy | Clinical Trial  Randomized Controlled Trial  Feasibility Studies |
| Embase | Psychosis  Schizoaffective psychosis  Depressive psychosis  Paranoid psychosis  Acute Psychosis  Affective Psychosis  Manic Psychosis  Schizophrenia  Paranoid schizophrenia  Delusion  Hallucination  Auditory hallucination  Visual hallucination  Mental disease  Thought disorder | N/A | Cognitive Therapy  Cognitive Behavioral Therapy  Behaviour Therapy | Clinical Trial  Randomized Controlled Trial  Controlled Clinical Trial  Feasibility Study |
| CINAHL | MH “Psychiatric Patients”  MH “Mental Disorders, Chronic”  MH “Mental Disorders”  MH “Schizophrenia”  MH “Schizophrenia, Treatment-Resistant”  MH “Schizoaffective Disorder”  MH “Paranoid Disorders”  MH “Hallucinations”  MH “Affective Disorders, Psychotic”  MH “Psychotic Disorders” | MH “Attitude” | MH “Acceptance and Commitment Therapy”  MH “Behavior Therapy”  MH “Cognitive Therapy” | RCT  MH “Pilot Studies”  MH “Randomized Controlled Trials”  MH “Clinical Trials” |
| Web of Science | N/A | N/A | N/A | N/A |

### Adapted Quality Assessment Tool

Effective Public Health Practice Project (EPHPP) Quality Assessment Tool for Quantitative Studies – Adapted Version

**COMPONENT RATINGS**

1. **SELECTION BIAS**

**(Q1) Are the individuals selected to participate in the study likely to be representative of the target population?**

1 Very likely

2 Somewhat likely

3 Not likely

4 Can’t tell

**(Q2) What percentage of selected individuals agreed to participate?**

1 80 - 100% agreement

2 60 – 79% agreement

3 less than 60% agreement

4 Not applicable

5 Can’t tell

| RATE THIS SECTION | STRONG | MODERATE | WEAK |
| --- | --- | --- | --- |
| See Dictionary | 1 | 2 | 3 |

**DICTIONARY: SELECTION BIAS**

*(Q1) Participants are more likely to be representative of the target population if they are randomly selected from a comprehensive list of individuals in the target population (score very likely). They may not be representative if they are referred from a source (e.g. clinic) in a systematic manner (score somewhat likely) or self-referred (score not likely).*

*(Q2) Refers to the % of subjects in the control and intervention groups that agreed to participate in the study before they were assigned to intervention or control groups.*

1. **STUDY DESIGN**

Indicate the study design:

1 Randomized controlled trial

2 Controlled clinical trial

3 Cohort analytic (two group pre + post)

4 Case-control

5 Cohort (one group pre + post (before and after))

6 Interrupted time series

7 Other specify ____________________________

8 Can’t tell

**Was the study described as randomized? If NO, go to Component C.**

No Yes

**If Yes, was the method of randomization described? (See dictionary)**

No Yes

**If Yes, was the method appropriate? (See dictionary)**

No Yes

| RATE THIS SECTION | STRONG | MODERATE | WEAK |
| --- | --- | --- | --- |
| See Dictionary | 1 | 2 | 3 |

**DICTIONARY: STUDY DESIGN**

In this section, raters assess the likelihood of bias due to the allocation process in an experimental study. For observational studies, raters assess the extent that assessments of exposure and outcome are likely to be independent. Generally, the type of design is a good indicator of the extent of bias. In stronger designs, an equivalent control group is present and the allocation process is such that the investigators are unable to predict the sequence.

Randomized Controlled Trial (RCT)

An experimental design where investigators randomly allocate eligible people to an intervention or control group. A rater should describe a study as an RCT if the randomization sequence allows each study participant to have the same chance of receiving each intervention and the investigators could not predict which intervention was next. If the investigators do not describe the allocation process and only use the words ‘random’ or ‘randomly’, the study is described as a controlled clinical trial.

See below for more details.

Was the study described as randomized?

Score YES, if the authors used words such as random allocation, randomly assigned, and random assignment.

Score NO, if no mention of randomization is made.

Was the method of randomization described?

*Score YES, if the authors describe any method used to generate a random allocation sequence.*

*Score NO, if the authors do not describe the allocation method or describe methods of allocation such as alternation, case record numbers, dates of birth, day of the week, and any allocation procedure that is entirely transparent before assignment, such as an open list of random numbers of assignments. If NO is scored, then the study is a controlled clinical trial.*

Was the method appropriate?

*Score YES, if the randomization sequence allowed each study participant to have the same chance of receiving each intervention and the investigators could not predict which intervention was next. Examples of appropriate approaches include assignment of subjects by a central office unaware of subject characteristics, or sequentially numbered, sealed, opaque envelopes.*

*Score NO, if the randomization sequence is open to the individuals responsible for recruiting and allocating participants or providing the intervention, since those individuals can influence the allocation process, either knowingly or unknowingly.*

*If NO is scored, then the study is a controlled clinical trial.*

Controlled Clinical Trial (CCT)

An experimental study design where the method of allocating study subjects to intervention or control groups is open to individuals responsible for recruiting subjects or providing the intervention. The method of allocation is transparent before assignment, e.g. an open list of random numbers or allocation by date of birth, etc.

Cohort analytic (two group pre and post)

An observational study design where groups are assembled according to whether or not exposure to the intervention has occurred. Exposure to the intervention is not under the control of the investigators. Study groups might be non- equivalent or not comparable on some feature that affects outcome.

Case control study

A retrospective study design where the investigators gather ‘cases’ of people who already have the outcome of interest and ‘controls’ who do not. Both groups are then questioned or their records examined about whether they received the intervention exposure of interest.

Cohort (one group pre + post (before and after)

The same group is pretested, given an intervention, and tested immediately after the intervention. The intervention group, by means of the pretest, act as their own control group.

Interrupted time series

A time series consists of multiple observations over time. Observations can be on the same units (e.g. individuals over time) or on different but similar units (e.g. student achievement scores for particular grade and school). Interrupted time series analysis requires knowing the specific point in the series when an intervention occurred.

Other:

One time surveys or interviews

1. **CONFOUNDERS**

**(Q1) Were there important differences between groups prior to the intervention?**

1 Yes

2 No

3 Can’t tell

4 Not applicable (See dictionary)

The following are examples of confounders:

1 Race

2 Sex

3 Marital status/family

4 Age

5 SES (income or class)

6 Education

7 Health status

8 Pre-intervention score on outcome measure

**(Q2) If yes, indicate the percentage of relevant confounders that were controlled (either in the design (e.g.**

**stratification, matching) or analysis)?**

1 80 – 100% (most)

2 60 – 79% (some)

3 Less than 60% (few or none)

4 Can’t Tell

| RATE THIS SECTION | STRONG | MODERATE | WEAK |
| --- | --- | --- | --- |
| See Dictionary | 1 | 2 | 3 |

**DICTIONARY: CONFOUNDERS**

*By definition, a confounder is a variable that is associated with the intervention or exposure and causally related to the outcome of interest. Even in a robust study design, groups may not be balanced with respect to important variables prior to the intervention. The authors should indicate if confounders were controlled in the design (by stratification or matching) or in the analysis. If the allocation to intervention and control groups is randomized, the authors must report that the groups were balanced at baseline with respect to confounders (either in the text or a table). If no control or comparison group was included in the study and the questions in this section do not apply, automatically rate the section as 3 WEAK and move on to section C.*

1. **BLINDING**

**(Q1) Was (were) the outcome assessor(s) aware of the intervention or exposure status of participants?**

1 Yes

2 No

3 Can’t tell

4 Not applicable

**(Q2) Were the study participants aware of the research question?**

1 Yes

2 No

3 Can’t tell

| RATE THIS SECTION | STRONG | MODERATE | WEAK |
| --- | --- | --- | --- |
| See Dictionary | 1 | 2 | 3 |

**DICTIONARY: BLINDING**

*(Q1) If no control or comparison group was included in the study, automatically rate the section as 3 WEAK and move onto section E. Otherwise, assessors should be described as blinded to which participants were in the control and intervention groups. The purpose of blinding the outcome assessors (who might also be the care providers) is to protect against detection bias.*

*(Q2) Study participants should not be aware of (i.e. blinded to) the research question. The purpose of blinding the participants is to protect against reporting bias.*

1. **DATA COLLECTION METHODS**

**(Q1) Were data collection tools shown to be valid?**

1 Yes

2 No

3 Can’t tell

**(Q2) Were data collection tools shown to be reliable?**

1 Yes

2 No

3 Can’t tell

| RATE THIS SECTION | STRONG | MODERATE | WEAK |
| --- | --- | --- | --- |
| See Dictionary | 1 | 2 | 3 |

**DICTIONARY: DATA COLLECTION METHODS**

*Tools for outcome measures of interest to the review must be described as reliable and valid. If ‘face’ validity or ‘content’ validity has been demonstrated, this is acceptable.*

Self reported data includes data that is collected from participants in the study (e.g. completing a questionnaire, survey, answering questions during an interview, etc.).

Assessment/Screening includes objective data that is retrieved by the researchers. (e.g. observations by investigators).

Medical Records/Vital Statistics refers to the types of formal records used for the extraction of the data.

*Reliability and validity can be reported in the study or in a separate study. For example, some standard assessment tools have known reliability and validity.*

1. **WITHDRAWALS AND DROP-OUTS**

**(Q1) Were withdrawals and drop-outs reported in terms of numbers and/or reasons per group?**

1 Yes

2 No

3 Can’t tell

4 Not Applicable (i.e. one time surveys or interviews)

**(Q2) Indicate the percentage of participants completing the study. (If the percentage differs by groups, record the**

**lowest).**

1 80 -100%

2 60 - 79%

3 less than 60%

4 Can’t tell

5 Not Applicable (i.e. Retrospective case-control)

| RATE THIS SECTION | STRONG | MODERATE | WEAK |
| --- | --- | --- | --- |
| See Dictionary | 1 | 2 | 3 |

**DICTIONARY: WITHDRAWALS AND DROP-OUTS**

Score YES if the authors describe BOTH the numbers and reasons for withdrawals and drop-outs.

Score NO if either the numbers or reasons for withdrawals and drop-outs are not reported.

Score NOT APPLICABLE if the study was a one-time interview or survey where there was not follow up data reported.

*The percentage of participants completing the study refers to the % of subjects remaining in the study at the final data collection period in all groups (i.e. control and intervention groups).*

1. **INTERVENTION INTEGRITY**

(**Q1) What percentage of participants received the allocated intervention or exposure of interest?**

1 80 -100%

2 60 - 79%

3 less than 60%

4 Can’t tell

**(Q2) Was the consistency of the intervention measured?**

1 Yes

2 No

3 Can’t tell

**(Q3) Is it likely that subjects received an unintended intervention (contamination or co-intervention) that may**

**influence the results?**

4 Yes

5 No

6 Can’t tell

**DICTIONARY: INTERVENTION INTEGRITY**

*The number of participants receiving the intended intervention should be noted (consider both frequency and intensity). For example, the authors may have reported that at least 80 percent of the participants received the complete intervention. The authors should describe a method of measuring if the intervention was provided to all participants the same way. As well, the authors should indicate if subjects received an unintended intervention that may have influenced the outcomes. For example, co-intervention occurs when the study group receives an additional intervention (other than that intended). In this case, it is possible that the effect of the intervention may be over-estimated. Contamination refers to situations where the control group accidentally receives the study intervention. This could result in an under-estimation of the impact of the intervention.*

1. **ANALYSES**

**(Q1) Indicate the unit of allocation (circle one)**

community organization/institution practice/office Individual

**(Q2) Indicate the unit of analysis (circle one)**

community organization/institution practice/office Individual

**(Q3) Are the statistical methods appropriate for the study design?**

1 Yes

2 No

3 Can’t tell

**(Q4) Is the analysis performed by intervention allocation status (i.e. intention to treat) rather than the actual**

**intervention received?**

1 Yes

2 No

3 Can’t tell

**DICTIONARY: ANALYSIS APPROPRIATE TO QUESTION**

Was the quantitative analysis appropriate to the research question being asked?

*An intention-to-treat analysis is one in which all the participants in a trial are analyzed according to the intervention to which they were allocated, whether they received it or not. Intention-to-treat analyses are favoured in assessments of effectiveness as they mirror the noncompliance and treatment changes that are likely to occur when the intervention is used in practice, and because of the risk of attrition bias when participants are excluded from the analysis.*

**COMPONENT RATINGS OF STUDY**

|  | **STRONG**  **1** | **MODERATE**  **2** | **WEAK**  **3** |
| --- | --- | --- | --- |
| **A SELECTION BIAS** |  |  |  |
| **B STUDY DESIGN** |  |  |  |
| **C CONFOUNDERS** |  |  |  |
| **D BLINDING** |  |  |  |
| **E DATA COLLECTION METHOD** |  |  |  |
| **F WITHDRAWALS AND DROP-OUTS** |  |  |  |

For each of the six components A – F, use the following descriptions as a roadmap.

**A) SELECTION BIAS**

Strong: The selected individuals are very likely to be representative of the target population (Q1 is 1) and there is greater than 80% participation (Q2 is 1).

Moderate: The selected individuals are at least somewhat likely to be representative of the target population (Q1 is 1 or 2); and there is 60 - 79% participation (Q2 is 2). ‘Moderate’ may also be assigned if Q1 is 1 or 2 and Q2 is 5 (can’t tell).

Weak: The selected individuals are not likely to be representative of the target population (Q1 is 3); or there is less than 60% participation (Q2 is 3) or selection is not described (Q1 is 4); and the level of participation is not described (Q2 is 5).

**B) DESIGN**

Strong: will be assigned to those articles that described RCTs and CCTs.

Moderate: will be assigned to those that described a cohort analytic study, a case control study, a cohort design, or an interrupted time series.

Weak: will be assigned to those that used any other method or did not state the method used.

**C) CONFOUNDERS**

Strong: will be assigned to those articles that controlled for at least 80% of relevant confounders (Q1 is 2); or (Q2 is 1).

Moderate: will be given to those studies that controlled for 60 – 79% of relevant confounders (Q1 is 1) and (Q2 is 2).

Weak: will be assigned when less than 60% of relevant confounders were controlled (Q1 is 1) and (Q2 is 3) or control of confounders was not described (Q1 is 3) and (Q2 is 4); or there was no control group (Q1 is 4).

**D) BLINDING**

Strong: The outcome assessor is not aware of the intervention status of participants (Q1 is 2); and the study participants are not aware of the research question (Q2 is 2).

Moderate: The outcome assessor is not aware of the intervention status of participants (Q1 is 2); or the study participants are not aware of the research question (Q2 is 2).

Weak: The outcome assessor is aware of the intervention status of participants (Q1 is 1); and the study participants are aware of the research question (Q2 is 1); or blinding is not described (Q1 is 3 and Q2 is 3); or there was no control group (Q1 is 4).

**E) DATA COLLECTION METHODS**

Strong: The data collection tools of interest have been shown to be valid (Q1 is 1); and the data collection tools have been shown to be reliable (Q2 is 1).

Moderate: The data collection tools have been shown to be valid (Q1 is 1); and the data collection tools have not been shown to be reliable (Q2 is 2) or reliability is not described (Q2 is 3).

Weak: The data collection tools have not been shown to be valid (Q1 is 2) or both reliability and validity are not described (Q1 is 3 and Q2 is 3).

**F) WITHDRAWALS AND DROP-OUTS - a rating of:**

Strong: will be assigned when the follow-up rate is 80% or greater (Q1 is 1 and Q2 is 1).

Moderate: will be assigned when the follow-up rate is 60 – 79% (Q2 is 2) OR Q1 is 4 or Q2 is 5.

Weak: will be assigned when a follow-up rate is less than 60% (Q2 is 3) or if the withdrawals and drop-outs were not described (Q1 is No or Q2 is 4).

Not Applicable: if Q1 is 4 or Q2 is 5.

**GLOBAL RATING FOR THIS PAPER (circle one):**

1 STRONG (no WEAK ratings)

2 MODERATE (one WEAK rating)

3 WEAK (two or more WEAK ratings)

With both reviewers discussing the ratings:

Is there a discrepancy between the two reviewers with respect to the component (A-F) ratings?

No Yes

If yes, indicate the reason for the discrepancy:

1 Oversight

2 Differences in interpretation of criteria

3 Differences in interpretation of study

**Final decision of both reviewers (circle one):**

| GLOBAL RATING | STRONG | MODERATE | WEAK |
| --- | --- | --- | --- |
| See Dictionary | 1 | 2 | 3 |

### Supplementary Figure 1: Funnel Plots

1. **BCSS Negative-self at End of Therapy**

1. **BCSS Negative-self at Follow up**

1. **BCSS Positive-self at End of Therapy**

1. **BCSS Positive-self at Follow-up**

1. **BCSS Negative-other at End of Therapy**

1. **BCSS Negative-other at Follow-up**

1. **BCSS Positive-other at End of Therapy**

1. **BCSS Positive-other at Follow-up**
